# High-Throughput Generation of Bipod (Fab × scFv) Bispecific Antibodies Exploits Differential Chain Expression and Affinity Capture

**DOI:** 10.1038/s41598-020-64536-w

**Published:** 2020-05-05

**Authors:** Thomas C. Nesspor, Kyle Kinealy, Nicholas Mazzanti, Michael D. Diem, Kevin Boye, Hunter Hoffman, Christine Springer, Justin Sprenkle, Gordon Powers, Haiyan Jiang, Sherry L. La Porte, Rajkumar Ganesan, Sanjaya Singh, Adam Zwolak

**Affiliations:** Biologics Discovery, Janssen Research & Development, LLC, Spring House, PA 19477 USA

**Keywords:** High-throughput screening, High-throughput screening, Preclinical research, Cancer, Immunosuppression, Antibody therapy, Biologics, Drug screening

## Abstract

Generation of bispecific antibodies (BsAbs) having two unique Fab domains requires heterodimerization of the two heavy chains and pairing of each heavy chain with its cognate light chain. An alternative bispecific scaffold (Bipod) comprising an scFv and a Fab on a heterodimeric Fc eliminates the possibility of light chain mispairing. However, unpredictable levels of chain expression and scFv-induced aggregation can complicate purification and reduce the yield of desired Bipod. Here, we describe a high-throughput method for generation of Bipods based on protein A and CH1 domain affinity capture. This method exploits over-expression of the scFv chain to maximize heterodimer yield. Bipods purified by this method have purity suitable for cell-based functional assays and *in vivo* studies.

## Introduction

Bispecific antibodies (BsAbs) have generated significant interest for therapeutic development due to their novel mechanisms of action. BsAbs can be used for immune cell redirection, targeting multiple antigens or epitopes on a single antigen, immune checkpoint modulation, or to enhance the payload delivery of antibody-drug conjugates, with the number of applications ever expanding and nearly one hundred BsAbs in clinical development^1–5^.

Numerous BsAb formats have been developed^6^, and they can be grouped into those lacking an Fc region and those having an Fc region. Although each format has specific advantages, BsAbs harboring an Fc region are more prominent in research and clinical settings^1^ and are often favored due to their long serum half-lives mediated by FcRn-based recycling and due to the ability of the Fc region to mediate effector functions^7^. The Fc region can also be “silenced” to prevent effector function when desired^8^. BsAbs harboring an Fc region can be generated by either adding an additional binding moiety, such as a single-chain fragment variable (scFv) onto either the N- or C-terminus of either the heavy chain (HC) or the light chain (LC) to generate a symmetric BsAb. Alternatively, an asymmetric BsAb can be generated by introduction of mutations in the HC CH3 domain, which forms most of the inter-chain contacts within the HC-HC interface, such that heterodimerization is favored over homodimerization. Asymmetric BsAbs are often advantageous over homodimeric BsAbs since they allow monovalent binding to each target. This is particularly important for T cell redirection approaches, since bivalent binding to T cells could lead to undesired activation and toxicity^1^.

Numerous sets of mutations that promote heterodimerization over homodimerization have been described^9–11^. In addition to HC heterodimerization, this approach necessitates a strategy to ensure proper pairing of the cognate light chains, and several solutions have been described. Introduction of complementary mutations in the HC-LC interface can drive proper pairing^12–14^. Other groups have used Fabs which share a common LC^15^, and still other groups have replaced one of the Fab arms with scFv or single domain Abs (VHH) to overcome the challenge of HC-LC pairing^16^. Asymmetric BsAb formats lend themselves well to immune cell redirecting BsAbs such as the ones described here due to the preference for monovalent immune cell binding, and relatively close distance between the immune cell and cancer cell targeting arm, which drives effective immune synapse formation.

Criteria for BsAbs suitable for clinical development include; relative ease of production, high stability, and favorable activity. To meet these criteria, many variables must be screened in a research setting, and therefore, methods to efficiently produce and screen large panels of high purity BsAbs are crucial. This is especially true for immune cell engaging BsAbs, as small amounts of contaminating homodimer can confound functional analysis. Here, we focus on a BsAb format, which we term a “Bipod”, in which one of the binding arms is a Fab while the other is a scFv. To generate these asymmetric BsAb, one HC contains T350V, L351Y, F405A, Y407V mutations and the other HC contains the complementary T350V, T366L, K392L, T394W mutations which have previously been shown to enhance heterodimerization^11,17^. We describe a novel method for high-throughput purification of bipods, with purity suitable for downstream functional assays.

## Results

### DNA transfection ratio

**Figure 1:**
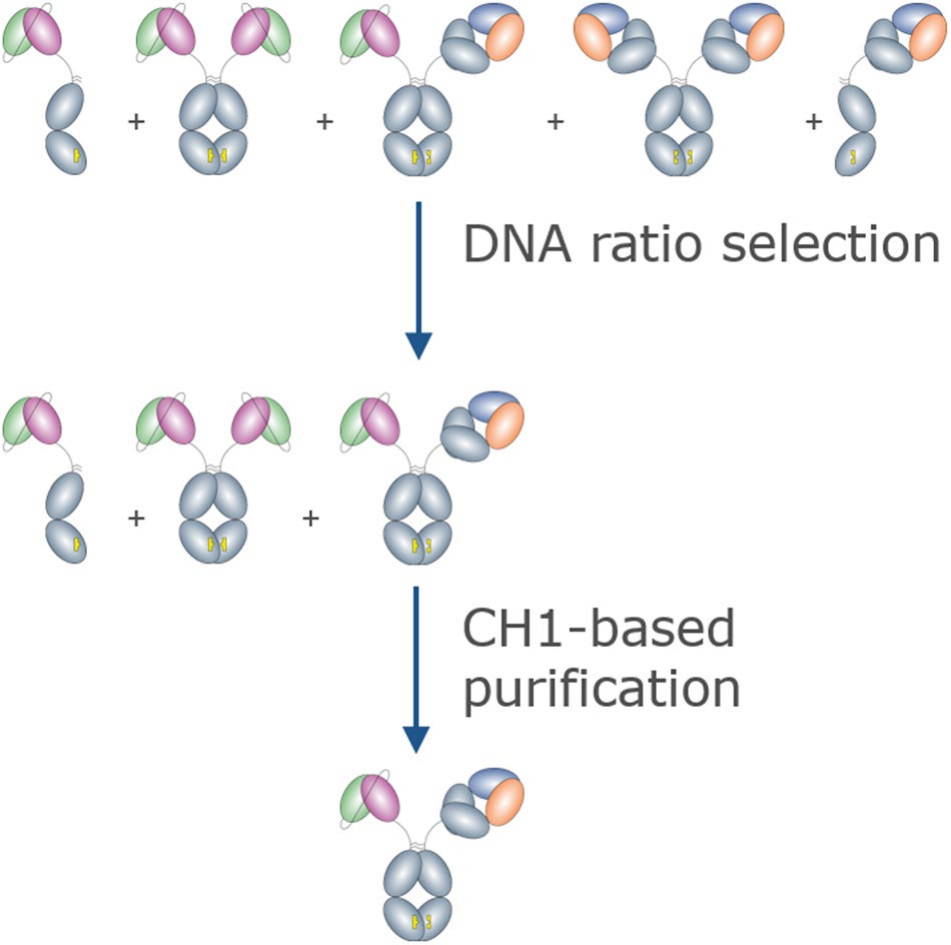
Cartoon illustration of the purification scheme which shows the potential species present after co-expression and purification of the “bipod” BsAb. Over-expression of the scFv-Fc arm saturates the HC-only species. Subsequent CH1-based purification selects only the desired heterodimeric BsAb.

We sought to identify a BsAb format and a method for generating BsAbs that would be suitable for high-throughput production of large panels, and which would result in highly pure molecules for functional and biophysical screening. We chose to use an asymmetric bispecific antibody comprising a full heavy chain paired with its cognate light chain on one subunit and an scFv fused to the Fc on the other subunit, since it eliminates the challenge of pairing two unique light chains with their appropriate heavy chains (Fig. 1). Each chain was expressed from its own plasmid having an identical promoter. The scFv-Fc and heavy chains featured complementary mutations designed to enhance heterodimerization described previously^11^. Briefly, the scFv-Fc chain (chain A) contained mutation of T350V, L351Y, F405A, Y407V and the heavy chain (chain B) contained mutation of T350V, T366L, K392L, T394W. Given equal expression of each heavy chain, these mutations in human IgG1 were shown to result in ~95% heterodimeric species having biophysical properties similar to a wild-type IgG1^11^. Expressed on its own, chain A exists as a population of ~90% half-Ab and 10% homodimer, whereas chain B is ~40% half-Ab and ~60% homodimer (Supplementary Fig. S1).

Light chain, which is required for heavy chain secretion^18^ was always over-expressed with respect to its cognate heavy chain. The scFv-Fc chain, heavy chain, and light chains were expressed using a DNA molar ratio of 2:1:3. This DNA ratio resulted in over-expression of the scFv-Fc chain in >95% of BsAbs tested. The excess scFv-Fc chain reduces the likelihood of unpaired Fab heavy chain or Fab-Fab heavy chain homodimer such that contaminants are limited to scFv-Fc only species (Supplementary Fig. S1).

### CH1-based purification of BsAbs

Culture supernatants from cells transfected using non-equimolar plasmid ratios contained a limited number of species: target BsAb, scFv-Fc monomer and homodimer, and LC homodimers. To purify the target BsAb we selected an IgG-CH1 affinity resin, because due to the lack of Fab-Fab homodimer, BsAb was the only species in the supernatant containing a CH1 domain. We first sought to identify a DNA ratio of chain A to chain B which would be sufficient to over-express the scFv-Fc chain in a majority of BsAbs. Since the expression level of each polypeptide is related to the sequence of the variable region and can vary by several fold, we tested whether DNA ratios of 1:1:3 or 2:1:3 scFv-Fc:HC:LC would result in excess scFv-Fc for most Abs using the same method (Table 1). For a panel of ten BsAbs, the main peak, consisting of target BsAb and scFv-Fc was ~67% for a DNA ratio of 1:1:3 compared to 81% for a DNA ratio of 2:1:3, suggesting that two-fold over-expression of the scFv-Fc chain would be generally sufficient to limit off-target species.

**Table 1:** Comparison of the % Main SEC peak at two different DNA ratios.

Sample	%Main peak	
	1:1:3	2:1:3
BsAb70	81	84
BsAb71	71	80
BsAb72	65	86
BsAb74	65	90
BsAb75	81	75
BsAb76	63	85
BsAb77	50	73
BsAb78	77	85
BsAb79	69	77
BsAb80	43	77

**Figure 2:**
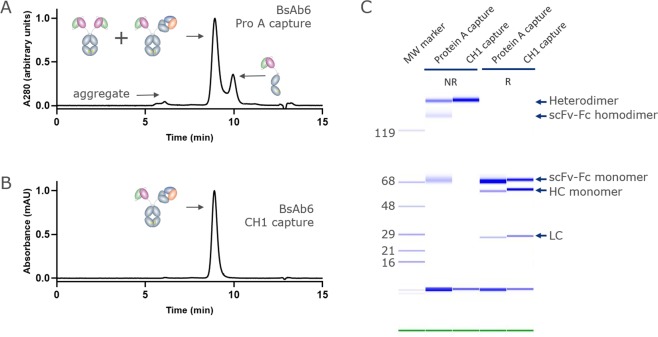
Example of a BsAb purified using the DNA imbalance + CH1 purification method. (**A**) Analytical SEC chromatogram of the initial protein A eluate featuring a main peak comprised of target heterodimer and scFv-Fc homodimer in addition to a minor peak comprised of scFv-Fc half Ab. (**B**) Analytical SEC chromatogram of the CH1 eluate from (**A**) showing capture of only the desired heterodimeric BsAb. (**C**) Non-reduced GXII analysis of the protein A eluate (lane 1) and the CH1 eluate (lane 2). Captured species are indicated on the gel image.

While IgG-CH1 capture could isolate the BsAb in a single step, we then sought to characterize the population of scFv-only species and target BsAb. Therefore, we first captured Fc-containing species using protein A affinity chromatography and analyzed the product using analytical size-exclusion chromatography (SEC) and capillary electrophoresis (Fig. 2, Table 2). The SEC chromatogram displayed 3 distinct peaks (Fig. 2A). A minor peak eluting at ~6 min contained aggregated species. The major peak, eluting at ~9 min contained the target BsAb and scFv-Fc-only homodimer, as confirmed by capillary electrophoresis (Fig. 2C). Another peak eluting at ~10 min contained scFv-Fc monomer. For this panel, the estimated % main peak ranged from 57 to 90% target species, based on quantitation of SEC (Fig. 2). The protein A eluate was then purified using CH1-affinity resin to remove species that lack a CH1 domain, or scFv-Fc only species, consistent with the capillary electrophoresis result (Fig. 2B,C). The resulting protein solutions were typically >97% target BsAb. One antibody, BsAb2, was only 91% pure after CH1 capture, and this may have been due to instability of the scFv moiety, which caused time-dependent aggregation with Fab-containing species. To confirm that the CH1 capture method could be used for different panels of BsAbs, we showed that another panel, purified in the identical process, produced similar levels of protein purity even at target species levels as low as 21% after protein A purification (Supplementary Table S1).

**Table 2:** Purity of BsAbs after protein A capture and a second CH1 affinity capture step.

Sample	Estimated % Main peak post protein A	Estimated % Main peak post CH1 capture
BsAb2	57.1	91.5
BsAb3	83.3	99.1
BsAb4	84.5	99.3
BsAb5	80.8	99.3
BsAb6	70.9	99.3
BsAb7	80.3	97.4
BsAb8	84.9	99.5
BsAb9	77.8	98.9
BsAb10	89.7	99.4
BsAb11	82.8	98.3

**Figure 3:**
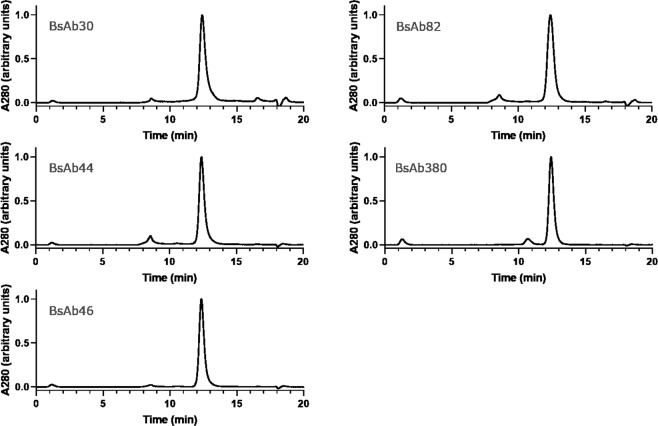
Examples of BsAbs purified using the direct, one-step CH1 purification method. Analytical SEC chromatograms of the CH1 eluate featuring a main peak comprised of target heterodimer and minor aggregate species.

We then asked whether “bipod” format BsAbs could be purified in one step using CH1 capture directly from culture supernatants. Five BsAbs were expressed using a 2:1:3 ratio of scFv-Fc:HC:LC and captured directly by CH1 affinity, and the product was analyzed by SEC (Fig. 3, Table 3). Because we have shown that CH1 affinity capture effectively eliminates scFv homodimer, we use main peak area as a surrogate for BsAb purity. The % purity of each BsAb ranged from 88 to 96% BsAb. The slightly lower % target species for BsAbs produced by direct CH1-based capture was generally due to aggregated protein, rather than excess homodimer or half-antibody. In general, BsAbs could be purified in one-step directly by CH1 capture yielding BsAbs >90% pure.

**Table 3:** Analysis of BsAb purity after one-step CH1 purification.

Sample	% BsAb
BsAb30	95.4
BsAb44	88.4
BsAb46	95.5
BsAb82	91.0
BsAb380	93.0

### HTP production of BsAbs using non-equimolar plasmid ratios

**Figure 4:**
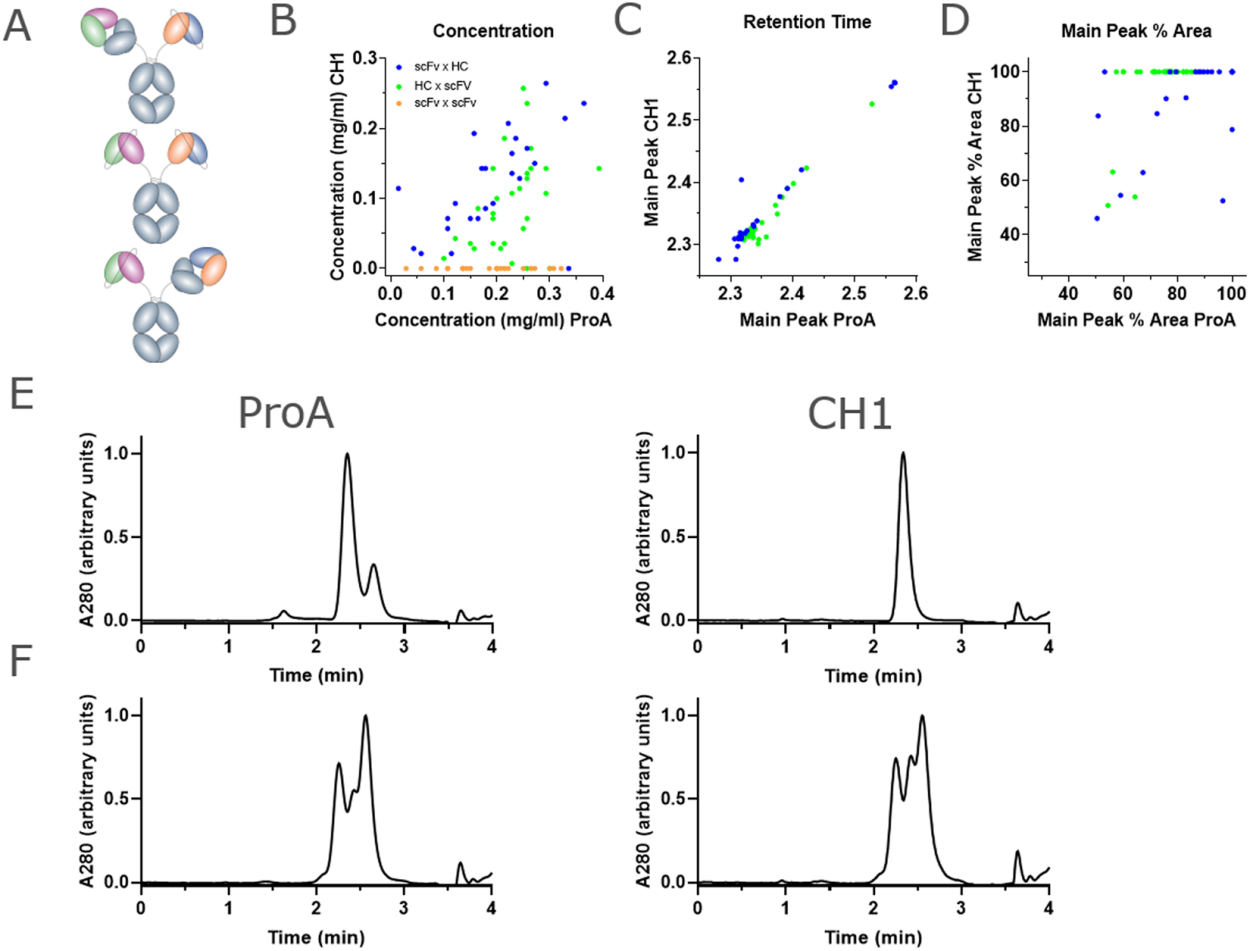
Analysis of BsAbs generated by DNA imbalance and CH1 capture using high-throughput methods. (**A**) Comparison of the total yield of antibody from CH1 capture vs protein A capture showing that over-expression of the scFv-Fc arm generally results in saturation of the HC arm, allowing CH1 capture of highly pure BsAb. The two “bipod” format BsAbs (HC × scFv-Fc or scFv × HC) are indicated in blue and green. scFv-Fc × scFv-Fc format bipods are shown in orange. (**B**) Comparison of the elution time of the main peak from CH1 capture vs protein A capture showing that the main peak is generally represented by target heterodimer and is consistent between methods. (**C**) Comparison of the % of the total protein population represented by the main peak from CH1 capture vs protein A capture showing that CH1 capture generally results in highly pure target BsAb. (**D**,**E**) Analytical SEC analysis of two proteins which illustrate the requirement for over-expression of the scFv-Fc arm. In D, excess DNA of the scFv-Fc arm results in over-expression of this component, leading to saturation of the HC-only species during expression. After protein A capture, CH1 capture retains only target BsAb. In E, over-expression of the HC component prevents its saturation with scFv-Fc arm, and prevents isolation of the target BsAb after protein A capture.

Since the CH1-based affinity purification of a small panel of BsAbs resulted in highly pure product, we then asked whether this method could be applied to larger panels of Abs produced at smaller scale in a high-throughput process. For this, we used a panel of 119 BsAbs having either the HC × scFv-Fc, scFv-Fc × HC, or scFv-Fc × scFv-Fc (Fig. 4A) format. The BsAbs in the panel were purified either by protein A or by CH1-capture, and the concentration of the eluate was measured for each method (Fig. 4B). As expected, a set of scFv-Fc × scFv-Fc control BsAbs did not bind the CH1 resin, and thus resulted in no captured protein. For the set of “bipod” molecules, protein A capture yielded higher quantities of protein since it captures all Fc-containing species, whereas CH1 capture yields were lower, ranging from ~0.1 mg/mL to 0.3 mg/mL in 400 uL with an average of 0.15 mg/mL. To determine the composition of the captured protein, we analyzed each protein by SEC. The major peak eluted at the same retention time with either resin (Fig. 4C). To determine whether CH1 capture resulted in higher purity, we then determined the percent of the total population represented by the major peak, which for Protein A could contain both scFv homodimer and BsAb, but for CH1 capture contains only BsAb. Indeed, CH1 capture resulted in a larger percentage of target BsAb, as represented by the main peak percent area. This increase in purity would have been more pronounced had we been able to quantitate the amount of bsAb in the protein A-captured protein. Moreover, in 44 of 59 “bipod” proteins, CH1 capture resulted in ~100% target BsAb (Fig. 4D). Note that the “bipod” panel contained both HC × scFv-Fc (scFv chain was constant) and scFv-Fc × HC (HC chain was constant) BsAbs. The CH1-based enrichment of target species for the HC × scFv-Fc format occurred in 29 of 34 proteins, whereas the enrichment for scFv-Fc × HC format occurred in only 14 of 25 proteins. This was likely due to the lower expression level, on average, of the panel of variable scFv chains in this format and could be overcome by further increasing the ratio of scFv-Fc. These results suggest that if the scFv-Fc chain is overexpressed, it would saturate all the HC and that CH1-based affinity capture could then selectively capture the target BsAb, whereas if the HC is over-expressed, the CH1-based capture will not result in highly pure BsAb. An example of each case highlights that over-expression of the scFv-Fc chain does in fact result in saturation of the HC, preventing HC homodimers and half-Abs (Fig. 4E,F).

### BsAbs display anti-tumor cytotoxicity

**Figure 5:**
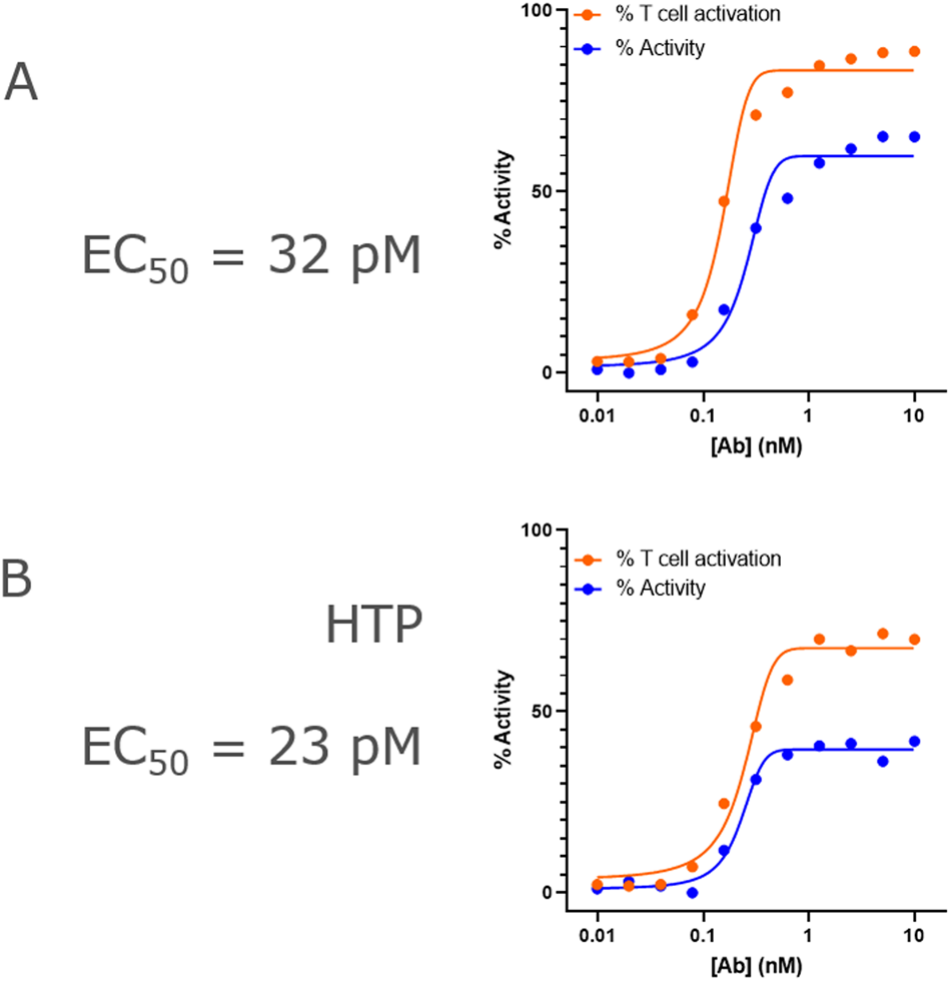
Functional comparison of a “bipod” BsAb produced using either large-scale (**A**) or high-throughput (**B**) methods. (**A**) Graph of the % CARNAVAL cell lysis vs concentration of antibody overlaid with the relative T cell activation, based on CD25 expression. The BsAb displays an EC_50_ for CARNAVAL cell killing of 32 pM. (**B**) The same experiment in (**A**) was performed using the BsAb generated in small scale using high-throughput methods, and it shows the antibody has similar activity, displaying an EC_50_ for CARNAVAL cell killing of 23 pM.

Cell-based cytotoxicity assays require highly pure protein to generate reliable results. The need for consistently high purity is especially true when screening large panels of bsAbs, where activity differences may be minor. We asked whether BsAbs generated using our HTP method would have comparable activity to the same BsAbs made at large scale. For this, we measured T cell-based cytotoxicity against CARNAVAL B cell lymphoma cells (Fig. 5A,B, Supplementary Fig. S3). The BsAb showed similar EC_50_ for target cell killing (0.032 nM vs 0.023 nM) when produced at either scale. The maximum cytotoxicity values were slightly different, and this was due largely to variability in cell cultures, since EC_50_ values were similar across BsAbs produced in either large scale or HTP scale.

**Figure 6:**
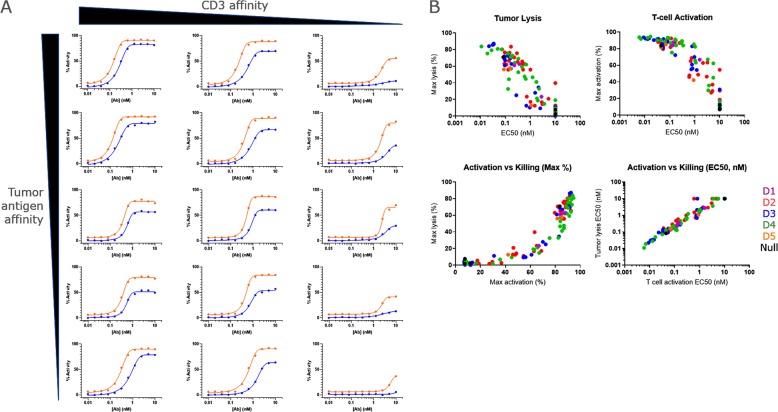
High-throughput generation of highly pure BsAbs allows interrogation of larger panels of BsAbs and analysis of wider functional questions. (**A**) Examples of CARNAVAL cell killing and T cell activation analysis using the same experiments as in (Fig. 4). BsAbs displayed a range of activities, from almost total cell killing, to total lack of activity. (**B**) The activities of a panel of BsAbs was analyzed and correlated with antigen binding epitope. Generation of large panels of highly pure, functional BsAbs allows interrogation of a range of antigen-binding affinities, epitopes, and format which generally present a technical challenge of purifying BsAbs since small levels of impurity can lead to large changes in activity.

To illustrate how functional characterization of high purity BsAbs produced using our HTP process can be used for lead selection, a panel of 59 BsAbs was generated. This panel used three different anti-CD3 v-regions to redirect T cells: a high affinity (K_d_ ~ 5 nM), medium affinity (Kd ~ 20 nM), and a low affinity (Kd ~ 300 nM). The redirection arms were paired with tumor-targeting v-regions with K_d_ ranging from 10 pM to 3 nM. These were formatted either as Fab × scFv-Fc or scFv-Fc × Fab Abs. The panel showed a range of activities, from >90% cytotoxicity to no activity (Fig. 6A). The cytotoxic activity of the BsAbs was highly dependent on affinity for CD3, affecting both the maximum cytotoxicity level and EC_50_ for killing (Fig. 6A**, rows**). Interestingly, modulation of the affinity of the targeting arm (from K_d_ = 50 pM to K_d_ = 2.9 nM) affected EC_50_ but had less effect on maximum cytotoxicity (Fig. 6A**, columns**).

In another example of how our process can enable early functional characterization of large BsAb panels, we generated a panel of BsAbs to evaluate the effects of epitope on T cell activation. For this panel of bipod BsAbs, tumor lysis and T cell activation, measured by CD25 expression, were inversely correlated with the EC_50_ for target cell binding (Fig. 6B). T cell activation was directly correlated to target cell lysis (Fig. 6B). The tumor antigen targeted by this panel was comprised of six domains (D1-D6), with D1 being membrane distal and D6 being membrane proximal. Most BsAbs bound to the central D2-D4 domains (Table 4), and analysis of the large number of binders allowed interrogation of whether targeting certain domains would result in more favorable activity. BsAbs that bound the centrally located D3 domain on the target cell antigen displayed a range of activities, but on average showed higher target cell lysis, and average cytotoxicity EC_50_ of than those that bound the adjacent D2 and D4 domains (Fig. 6B, Table 4), suggesting that D3-binding BsAbs may be more desirable than those that bound D4. Overall, T cell activation, based on CD25 expression, appeared to be correlated with maximum and EC_50_ for cytotoxicity (Fig. 6B). This example clearly illustrates how screening of multiple variables for a large panel of BsAbs, enabled by our HTP process, provided critical data for the early selection of lead candidates.

**Table 4:** Comparison of Activity of BsAbs.

Binding Domain	No. Abs	Avg. Kd (nM)	Average Cytotoxicity (EC50, nM)	Average max. cytotoxicity (%)	Avg. T cell activation (EC50, nM)	Average max. T cell activation (%)
1	1	1.6	0.15	58	0.1	85
2	12	1.1	0.76	58	0.7	82
3	10	0.41	0.22	64	0.1	88
4	12	0.95	0.70	57.1	0.3	85
5	0	NA	NA	NA	NA	NA
6	1	3.5	0.36	63	0.15	88

BsAbs that redirect T cells against target cells are often associated with high toxicity in clinical studies, and this toxicity is thought to be related to high relative affinity for T cells over target cells. Desirable T cell-redirecting BsAbs would display high cytotoxicity coupled with low T cell activation. Alternatively, some BsAbs may mediate target cell killing in the absence of T cell activation or effector function, such as those that can block signaling; for example, anti-EGFR Abs^19^. Thus, we measured activation of T cells, using CD25 expression as a confirmation of the mechanism of cytotoxicity, and to confirm T cell activation was not due to aggregation of BsAbs, leading to T cell crosslinking.

## Discussion

The method described here combines three elements to allow for purification of BsAbs having the asymmetric “bipod” format. First, at least one v-region must be stable as a scFv. In general, this requires pre-screening for stability prior to formatting into a BsAb, since aggregated species will not be efficiently purified away from target species. Other moieties that lack a CH1 domain, such as a VHH or antigen fusion, would also lend themselves to this purification method. Second, this method depends on limiting the expression of Fab arm-containing species thereby “steering” the product towards scFv contaminants, which lack a CH1 domain. We found that a scFv-Fc to HC ratio of 2:1 resulted in over-expression of the scFv-Fc chain in most cases. For those pairs having unusually high expressing HC, or low expressing scFv, further increases in DNA ratio may be needed to achieve high purity. Third is the use of CH1-affinity chromatography to isolate highly pure target BsAb beyond what can be achieved by protein A capture alone. For the panels tested, application of all three of these elements could routinely achieve 95% purity.

This method was applied for expression at the 2 mL scale to allow for production of large panels of BsAbs, and due to practical limitations of CH1 affinity resin. One theoretical limitation of our method is the necessarily lower yields due to over-expression of one subunit However, in an HTP format, where only small amounts of product are needed for screening, this is not of primary concern.

For single step purification, the imbalanced DNA ratio and CH1 purification steps result in highly pure target BsAb, although a limitation is the necessarily lower yields due to over-expression of one subunit. An alternative approach is to introduce into the CH1-containing subunit mutations that disrupt protein A binding, such as those described previously^20,21^. BsAbs designed in this way allow purification specific to each chain – protein A for the scFv-Fc chain or CH1 for the HC + LC. This approach allows the DNA for each HC to be transfected at 1:1 molar ratio. Panels of proteins produced in this way would then be subjected to both protein A and CH1-based affinity purification to remove contaminants solely containing either CH1 or wild-type Fc.

Protein A-based affinity purification of Abs is generally preferred in scientific settings due to its high specificity, ease of capture and elution, availability of GMP-quality resin, and its ability to be continually recycled via sodium hydroxide regeneration. During development of this method, we noted that proteins captured directly from media supernatant onto CH1 resin could be efficiently eluted. However, the standard wash with 20 mM sodium hydroxide resulted in retained endotoxin contamination in BsAbs purified using regenerated resin. Thus, column regeneration was performed with 40 mM NaOH. Additionally, multiple purifications performed on regenerated columns showed successively lower yields. This may have been due to degradation of the CH1 affinity matrix or to accumulation of an unknown media component which led to lower binding capacity in subsequent purifications. For this reason, in large scale batches, we often perform a first step purification with protein A to remove the media supernatant component.

Large panels of BsAbs are needed to explore the complex design space around BsAbs that includes affinity, valency, epitope and format. The process described here is amenable to high-throughput production to support the optimization of BsAbs. In functional screening, BsAbs purified using our HTP method retained similar activity to the same BsAb purified at larger scale. These analyses will improve lead molecule selection for therapeutic programs.

## Methods

### Protein expression and purification

#### High-throughput

Antibody scFv-Fc, HC, and LC were cloned into single-gene plasmids each having an identical CMV promoter. Plasmids were transfected into ExpiCHO cells (ThermoFisher) at a ratio of 2:1:3 of scFv-Fc:HC:LC, according to the manufacturer’s protocol. Cells were pelleted by centrifugation at 850 × g for 15 minutes and the culture supernatants were harvested. Plate based purification was performed in 2 mL 96-well 1 μm glass filter plates prepared in-house. Supernatants were applied to either MabSelect SuRe LX resin (GE Healthcare) or CaptureSelect IgG-CH1 Affinity Matrix (Thermo Fisher), eluted according to the manufacturers’ protocols and neutralized with Tris-HCl.

Neutralized eluates (0.5 mL) containing purified BsAbs were dialyzed into PBS, pH 7.2 and filtered using a 1 mL 96-well 0.2 μm Supor plate (Pall) prior to analysis. Quantification of each dialyzed elution pool was determined by measuring the absorbance at 280 nm using the Trinean DropSense (Unchained Labs). Purity of antibody solutions was assayed by analytical size-exclusion chromatography (SEC) using an Agilent AdvanceBio SEC. 300 A (Agilent) connected to an Agilent 1200 HPLC to monitor absorbance at 280 nanometers.

#### Large scale

Antibody scFv-Fc, HC, and LC were cloned into single-gene plasmids each having an identical CMV promoter. Plasmids were transfected into ExpiCHO cells (ThermoFisher) at a ratio of 2:1:3 of scFv-Fc: HC:LC, according to the manufacturer’s protocol. Cells were pelleted by centrifugation at 4000 × g for 12 minutes and the culture supernatants were harvested. Supernatants were applied to either mAbSelect Sure resin (GE Healthcare) or CaptureSelect CH1-XL affinity matrix (ThermoFisher) and eluted according to the manufacturers’ protocols.

Eluates containing purified BsAbs were dialyzed into 1 × PBS, pH 7.2 prior to analysis. Purity of antibody solutions was assayed by analytical size-exclusion chromatography (SEC) using a BioAssist G3SW_XL_ column (Tosoh). Capillary electrophoresis was performed using a Caliper LapChip GX II instrument according to the manufacturer’s protocol. CH1-XL resin was regenerated by incubation with 40 mM NaOH for 30 min.

#### Functional analysis

BsAbs were diluted to 40 nM in assay media (RPMI + 10% FBS HI + 1% Penicillin). Proteins were prepared in 3-fold serial dilutions for an 11-point titration in assay media in separate v-bottom polypropylene plates. CARNAVAL cells were diluted to 2 × 10^5^ cells/ml and plated at 20,000 cells/well in 100 mL of assay media. T cells were diluted to 2 × 10^6^ cells/mL and added to each well at 100,000 in 50 mL of assay media to the assay plates containing tumor target cells. 50uL of serially diluted BsAb was added to the well starting from 10 nM BsAb with 2-fold dilutions. Plates were incubated at 37 °C, 5% CO2 in a humidified cell culture incubator for 48 hours. After incubation, plates were centrifuged at 300 × g for 5 min to remove supernatants. Cells were washed by adding 150 uL of staining buffer and spun again at 300 × g for 5 min. Wash was repeated in the same manner by adding 200 uL of staining buffer.

Staining solution mixture contained: APC-conjugated anti-Human CD4 (1:500) (Thermofisher cat. # MHCD0405), APC-conjugated anti-Human CD8 (1:500) (Thermofisher cat. # MHCD0805), and Brilliant Violet 421-conjugated anti-Human CD25 (1:500) (Biolegend cat. # 302630). 50uL/well of staining solution mixture was added to the assay plates and incubated at room temperature for 20 min in the dark. 150uL staining buffer was added to all wells and wash twice by centrifugation at 400 × g for 3 min followed by removal of the supernatant. Vybrant DyeCycle Green (Thermofisher cat. # V35004) was prepared at 1:25 k in Intellicyt running buffer. Cell pellets were resuspended in 20 uL Intellicyt running buffer containing Sytox green live/dead stain (1:1000) or Vybrant DyeCycle Green, and plates were analyzed on the iQue PLUS Screener.

#### Gating strategy

A cell population was selected on FSC-H vs SSC-H, then T cell and tumor cell populations were selected on APC (RL1) vs SSC-H (Supplementary Fig. S3). Live and dead populations (live/dead stain) for both tumor and T cells from their respective dot plots on FSC-H vs Sytox Green (BL2) were selected. The live T cell population was used to gate activated T cell (CD25+) population on FSC-H vs Brilliant Violet (VL1). Gates were defined from control sample wells containing T-cells and tumor cells alone and in a mixture lacking BsAb. ForeCyt advanced metrics were used to calculate Tumor cell death as a percentage of dead Tumor cell events within tumor cell events, T cell viability as a percentage of viable T cell events within T cells events, and Activated T cells as a percentage of Activated T cells events within viable T cells events.

## Supplementary information

Supplementary Information.
