# Supplementary material for: High-Throughput Generation of Bipod (Fab × scFv) Bispecific Antibodies Exploits Differential Chain Expression and Affinity Capture
Source: Sci Rep. 2020 May 5;10:7557. doi: 10.1038/s41598-020-64536-w (PMC7200789; doi:10.1038/s41598-020-64536-w)
Supplement: Supplementary file 1 — Supplementary Information. [file 41598_2020_64536_MOESM1_ESM.docx]

**Supplementary Information for:**

**High-Throughput Generation of Bipod (Fab x scFv) Bispecific Antibodies Exploits Differential Chain Expression and Affinity Capture**

Thomas C. Nesspor [1], Kyle Kinealy [1], Nicholas Mazzanti [1], Michael D. Diem [1], Kevin Boye [1], Hunter Hoffman [1], Christine Springer [1], Justin Sprenkle [1], Gordon Powers [1], Haiyan Jiang [1], Sherry L. La Porte [1], Rajkumar Ganesan [1], Sanjaya Singh [1], Adam Zwolak* [1]

[1] Biologics Discovery, Janssen Research & Development, LLC, Spring House, PA, 19477, USA.

*Correspondence to [azwolak1@its.jnj.com](mailto:azwolak1@its.jnj.com)

| **Supplementary Table S1. Purity of BsAbs after protein A capture and a second CH1 affinity capture step** | | |
| --- | --- | --- |
| **Sample** | **Estimated %**  **Main peak post protein A** | **Estimated %**  **Main peak post CH1 capture** |
| BsAb910 | 55.88 | 99.35 |
| BsAb923 | 33.81 | 100.00 |
| BsAb924 | 27.7 | 100.00 |
| BsAb925 | 23.11 | 100.00 |
| BsAb931 | 21.47 | 100.00 |
| BsAb935 | 80.47 | 98.20 |
| BsAb937 | 67.61 | 98.65 |
| BsAb939 | 26.69 | 100.00 |
| BsAb941 | 87.35 | 97.67 |
| BsAb943 | 90.53 | 100.00 |

**
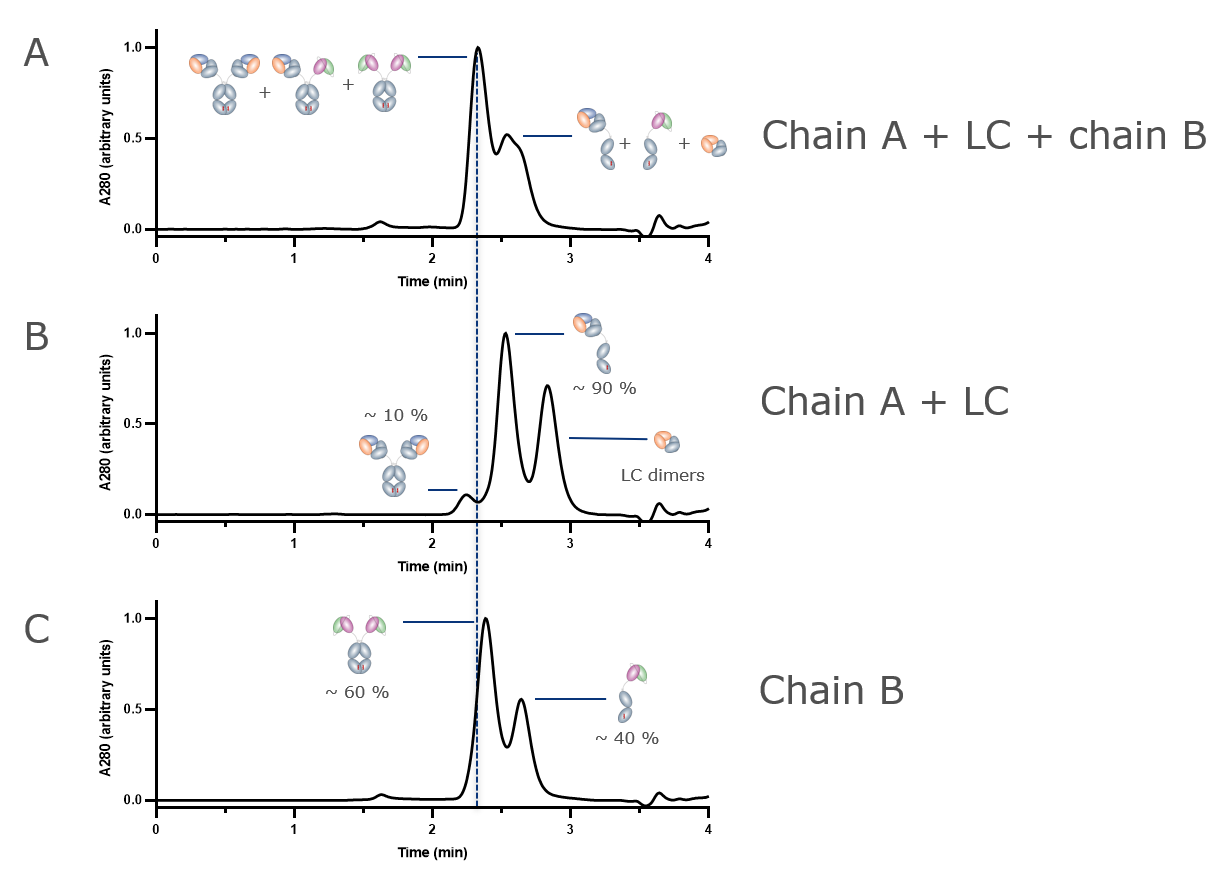
**

**Supplementary Figure S1.** Analysis of the propensity of Fc variant Abs to form homodimer or half-antibody by analytical SEC. (A) Chromatograph showing the total captured eluate from co-expression of a HC1 + LC + HC2 “bipod” bispecific antibody. Species are indicated on the graph. (B) Expression of HC1 + LC alone at 1:3 DNA ratio. (C) Expression of HC2 (scFv-Fc) alone.


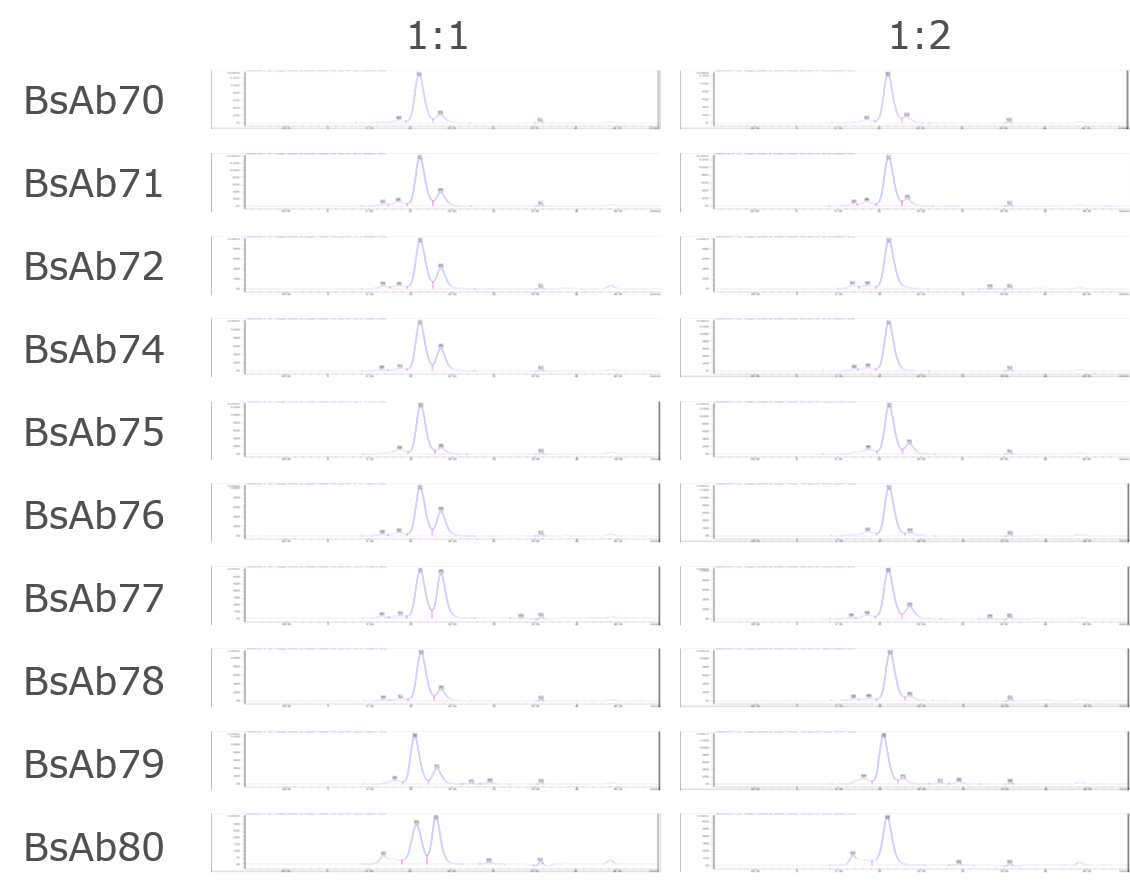


**Supplementary Figure S2.** Original capillary electrophoresis image used to generate the image in Figure 2. Image was cropped post generation only to remove antibody identities, without contrast manipulation during preparation of the figure.


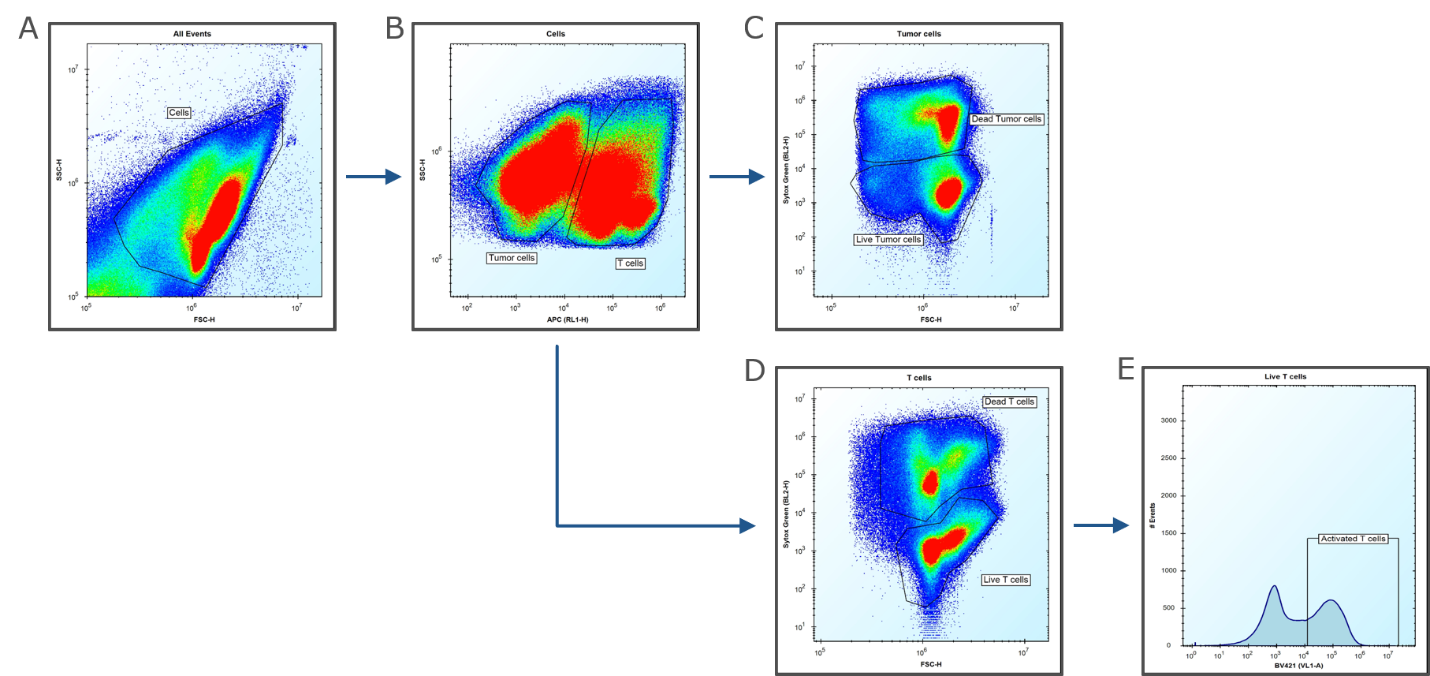


**Supplementary Figure S3.** Representative gating strategy for measuring bispecific antibody-mediated T cell-redirected functional activity. Cell viability and T cell activation each calculated as the percentage of events in terminal gate / events in parent gate. (A) gating of cells from all recorded events. (B) Gating of T cells (CD4^+^CD8^+^) vs target cells (unstained). (C) Gating of live vs dead target cells. (D) Gating of live vs dead T cells. (E) Gating of activated T cells (CD25^+^).
